# Lyophilized platelets inhibit platelet aggregation with simultaneous paradoxical promotion of platelet adhesion

**DOI:** 10.3389/fbioe.2022.941817

**Published:** 2022-08-19

**Authors:** Brian Schnoor, Anne-Laure Papa

**Affiliations:** Department of Biomedical Engineering, The George Washington University, Washington, DC, United States

**Keywords:** platelets, lyophilized platelets, platelet aggregation, platelet adhesion, blood product

## Abstract

Lyophilized platelets have been explored as a potential hemostatic agent due to their long-term ambient storage capabilities that make them readily available in various scenarios. Additionally, their high biocompatibility and the key role of platelet interactions in various clinical conditions make them a promising platform for drug delivery. To explore these applications and for wider clinical deployment, the interactions between lyophilized platelets and fresh platelets must be examined. This project characterized receptor expression on the lyophilized platelet surface and their ability to bind fibrinogen using flow cytometry. The effect of lyophilized platelets on aggregation of unaltered platelets was assessed using light transmission aggregometry while the effect on adhesion was evaluated using static and microfluidic assays. Lyophilized platelets maintained significant levels of GPIIb and GPVI receptors on their surface, though the expression was reduced from fresh platelets. Additionally, lyophilized platelets maintained GPIb expression similar to fresh platelets. Furthermore, 15.8% of the lyophilized platelets exhibited the active conformation of the GPIIb/IIIa receptor, indicating a significant increase over fresh platelets. Lyophilized platelets also exhibited an increase in exposed phosphatidylserine and fibrinogen binding. Despite the effect of lyophilized platelets in promoting the adhesion of fresh platelets on a collagen-coated surface, their net effect was inhibitory on platelet aggregation. This study demonstrates that lyophilized platelets can have paradoxical effects on platelet adhesion and aggregation, which could have an impact for clinical applications. Detailed characterization and engineering of these effects will be important for their continued development as a drug delivery platform.

## 1 Introduction

Lyophilized platelet (LP) formulations represent a promising platform for the development of a variety of therapeutic approaches. Most research to date has focused on LPs as a potential therapy for treating hemorrhage (Alemany et al., 1997; Bode et al., 1999; Fischer et al., 2002; Barroso et al., 2018; Bynum et al., 2019), because they present several potential advantages over traditional platelet transfusion. Fresh platelet transfusions are currently a widely used and essential therapy for preventing and treating hemorrhage (Stroncek and Rebulla, 2007). The effectiveness of this treatment method has long been recognized and existing clinical criteria are used to determine optimal use of platelet transfusions (Kumar et al., 2015; Stolla et al., 2015). However, there are some key weaknesses of current fresh platelet transfusions that LPs may be able to overcome. Under current methods, platelets can only be stored for 5 days prior to transfusion (Food and Drug Administration, 2020). Additionally, these platelet units must be kept under constant agitation at 20–24°C (Food and Drug Administration, 2020). In contrast, LPs can be stored for extended periods of time at room temperature and without the need for agitation. Since the LPs are stored in dry form, they are less susceptible to bacterial contamination during the storage phase. Additionally, the profound blood shortage noted in the ongoing COVID-19 pandemic have emphasized the critical need for the development of storable blood products (Al Mahmasani et al., 2021). These advantages of LPs could vastly increase the availability and utility of platelets for any number of treatments including hemostatic or drug delivery applications, and for wide capability deployment including resource limited conditions. A special emphasis has also been on the study of their circulation time and biodistribution in relevant models (Jackson et al., 1959; Fischer et al., 2001; Macko et al., 2016). This research has shown that LPs are rapidly cleared by the splenic macrophages (Fischer et al., 2001), possibly leading to their short circulation time and limited hemostatic effects in some models (Jackson et al., 1959; Macko et al., 2016). Thus, the LP approach may not be best suited for an application requiring extended circulation, but these LP carriers may be ideal for applications where the effect is intended for a more acute timescale.

The properties of these LPs also potentially create opportunities for drug delivery beyond hemorrhage and trauma. If LPs can be incorporated into a growing clot, they could potentially be leveraged as a fibrinolytic delivery platform to target thrombosis while mitigating the side effects of the loaded therapeutic drug, *via* a targeting effect. Furthermore, platelets have been shown to extensively interact with cancer cells (Gay and Felding-Habermann, 2011; Papa et al., 2019). LPs could use this bioaffinity to target the cancer cells or inhibit the cancer cell-platelet interactions *via* drug delivery/release.

Studies examining LP systems have covered many different formulations designed to maximize effectiveness. The most common formulations included platelets fixed with paraformaldehyde and then lyophilized in a solution of serum albumin (Jackson et al., 1959; Bode et al., 1999; Fischer et al., 2002; Valeri et al., 2004). Other formulations have used trehalose as a cryoprotectant (Bynum et al., 2019), or lyophilized the platelets without a cryoprotectant (Alemany et al., 1997; Fischer et al., 2001). These studies have assessed the effectiveness of the LPs for adhering to a thrombogenic surface and reducing bleeding in thrombocytopenic models. This foundational research has even led to clinical trials such as the ones developed by Cellphire (Cellphire Therapeutics, Inc., 2017; Cellphire Therapeutics, Inc., 2019; Cellphire Therapeutics, Inc., 2020; Cellphire Therapeutics, Inc., 2021; Cellphire Therapeutics, Inc., 2022). This research included a safety evaluation that showed no serious adverse effects after infusion of autologous LPs in healthy patients (NCT02223117 phase I) (Cellphire Therapeutics, Inc., 2017). Other trials currently underway are evaluating the safety and efficacy of allogenic LP formulations in thrombocytopenic (NCT03394755 phase I, NCT04631211 phase II) (Cellphire Therapeutics, Inc., 2019; Cellphire Therapeutics, Inc., 2021), and bleeding patients (NCT04631211 phase II, NCT04619108 Expanded Access) (Cellphire Therapeutics, Inc., 2020; Cellphire Therapeutics, Inc., 2021). Lastly, LP formulations are being evaluated to control bleeding during cardiopulmonary bypass surgery compared to platelets (NCT04709705 phase II) (Cellphire Therapeutics, Inc., 2022).

In order to develop these LPs into drug delivery systems though, the interactions between LPs and fresh platelets must be further characterized to best leverage this platform. Current research has explored many aspects of how LPs react independently of native platelets or under thrombocytopenic conditions. However, this study aims to bring new insight into how these LPs interact with and affect fresh platelet function. Additionally, understanding these interactions will help determine the best suited therapeutic applications for the LPs. Depending on the interactions, LPs can be further biofunctionalized and engineered to be used therapeutically to promote or inhibit specific platelet functions. However, all these applications depend on a robust understanding of how LPs impact the functioning of untreated native platelets.

The characterization of LPs in comparison to unaltered platelets was conducted by analyzing key receptors involved with platelet interactions. Then LPs were examined for the exposure of phosphatidylserine on the platelet surface, as well as the ability to bind fibrinogen. Subsequently, the analysis of the interaction of lyophilized and fresh human platelets *ex vivo* was assessed through the aggregation and adhesion of human platelets incubated with LPs. These experiments expand current understanding on how LPs interact with unaltered platelets. In brief, flow cytometry analysis demonstrates that LPs maintained significant levels of the key adhesion receptors GPIIb, GPVI, and GPIb. Additionally, a significant portion of LPs also expressed an activated form of the GP IIb/IIIa receptor, as well as increased phosphatidylserine exposure and fibrinogen binding. Finally, LPs demonstrated contrary effects on platelet function by inhibiting platelet aggregation but promoting platelet adhesion.

## 2 Materials and methods

### 2.1 Materials

The adenosine diphosphate (ADP) used for aggregation analysis was purchased from Chrono Log (cat# 384). The reagents for the lactate dehydrogenase (LDH) assay were obtained from Promega (cat# J2380). The dye-conjugated antibodies were purchased from BD Biosciences: APC mouse anti-human CD41a (cat# 559777), APC mouse IgG, ĸ isotype control (cat# 555751), FITC mouse anti-human PAC-1 (cat# 340507), FITC mouse IgM, ĸ isotype control (cat# 551448), FITC mouse anti-human CD42b (cat# 555472), FITC mouse IgG1, k isotype control (cat# 555748), PE mouse anti-human platelet GPVI (cat# 565241), and PE mouse IgG1, k isotype control (cat# 554680). Additionally, APC-labeled annexin V was obtained from Biolegend (cat# 640932). Alexa Fluor 647-conjugated fibrinogen was obtained from Thermo-Fisher Scientific (cat# F35200). Calcein AM was purchased from ThermoFisher (cat# C3099).

### 2.2 Lyophilized platelets and blood sources

LPs were purchased from Chrono-Log (cat# 299-2). The LPs are fixed with paraformaldehyde before lyophilization. Human whole blood was purchased from BioIVT, following approval from the Institutional Biosafety Committee at the George Washington University, and drawn with 3.8% sodium citrate. Commercial blood was collected as late as possible in the day and shipped overnight before being processed in our experiments. Untreated platelets were obtained in platelet rich plasma (PRP) *via* centrifugation at 150 g from the whole blood from healthy donors. Platelet poor plasma (PPP) was obtained *via* centrifugation of the remaining blood fraction after the PRP had been removed. If more PPP was required than could be obtained with this method, addition PPP was produced by centrifuging a portion of PRP at 2,000 g.

### 2.3 Flow cytometry for receptor characterization

In order to characterize the receptor availability, the lyophilized and untreated platelets were analyzed *via* flow cytometry. In brief, the platelets, lyophilized and untreated, were each stained for APC-CD41a (GPIIb), FITC-PAC-1 (specific to the activated GP IIb/IIIa complex), FITC-CD42b (GPIb), and PE-anti-GPVI. The staining for all receptors was conducted using 500,000 platelets or LPs in 50 uL of tyrode solution (136 mM NaCl, 12 mM NaHCO3, 2.9 mM KCl, 0.34 mM Na2H2PO4, 1 mM MgCl2, and 10 mM HEPES) and incubated for 20 min. The CD41a, PAC-1, and CD42b antibodies were added at a 1:50 dilution from the stock, while the GPVI antibody was added at a 2:25 dilution. Following the staining, the platelets were analyzed with the Novocyte flow cytometer (Agilent) with the 488 and 640 nm lasers activated and the appropriate detection filters. The platelets were then gated according to FSC (forward scatter) and SSC (side scatter). Then the median fluorescence and the percentage of stained platelets could be determined. Fluorescently conjugated IgG or IgM isotypes were used as controls.

### 2.4 Flow cytometry for phosphatidylserine exposure and fibrinogen binding

The exposure of phosphatidylserine on the surface of the lyophilized and fresh platelets was evaluated using flow cytometry. The lyophilized and fresh platelets were stained using APC-labeled annexin-V and analyzed with the Novocyte flow cytometer. The staining was conducted with 500,000 platelets or LPs in 100 uL of tyrode and incubated for 20 min. The annexin-V was added at a 1:25 dilution from the stock. To assess their fibrinogen binding, 500,000 of the lyophilized and fresh platelets were also separately incubated with 100 ug/mL Alexa Fluor 647-conjugated fibrinogen (2ug total) for 20 min in 20 uL of tyrode. The samples were then fixed with 1% paraformaldehyde and analyzed with the Novocyte flow cytometer. For both experiments the 640 nm laser was used and the platelets were gated according to FSC and SSC. The median fluorescence and the percentage of the platelets stained were determined.

### 2.5 Light transmission aggregometry

The aggregation of the lyophilized and untreated platelets was assessed using light transmission aggregometry (LTA) with a Model 490 4 + 4 instrument from Chrono-Log. Untreated platelet concentrations of 200,000; 150,000; 100,000; and 75,000 platelets/uL were prepared in plasma to represent a range of healthy and thrombocytopenic conditions. The aggregation of each suspension was measured in the presence of ADP as an agonist in concentrations of 20 uM and 50 uM. The 20 uM ADP concentration was selected based on previous work by Valeri *et al.* and Fitzpatrick *et al.* analyzing LP aggregation (Valeri et al., 2004; Fitzpatrick et al., 2013). The 50 uM ADP concentration was selected to assess the robustness of the antiplatelet effect in supra-physiological conditions. The experiments were repeated with a 5:0, 5:1, and 5:2 ratio of untreated platelets to LPs for each of the above concentrations (Supplementary Table S3). These ratios were selected to approximate the ratios achieved with one and two units of standard platelet transfusion. The volume of the mixture was 250 uL for each condition. The same concentration of LPs in PPP was used to produce an accurate reference sample. An additional trial with only LPs was conducted at each concentration as a control to verify that LPs do not undergo aggregation when exposed to ADP.

### 2.6 Platelet static adhesion assay

A procedure based around a LDH assay was developed to assess the adhesion of mixed lyophilized and untreated platelets on collagen. First a 1 mg/ml fibrillar collagen solution (Chrono-Log, cat# 385) was diluted with phosphate buffered saline (PBS) to 0.1 mg/ml and incubated in each well in 96-well plates for 1 h to form a collagen layer to which platelets could adhere. The wells were then gently rinsed with PBS to remove excess collagen. Then each mixture of lyophilized and untreated platelets was incubated in the wells for 2 h to allow adhesion to occur. Samples at 200,000 platelets/uL and 75,000 platelets/uL in plasma were used to represent healthy and thrombocytopenic conditions, respectively. For each respective condition an untreated platelets only, a LP only, a 5:1 platelet to LP, and a 5:2 platelet to LP trial was performed (Supplementary Table S3). After the incubation time, the suspension was removed, and the well was rinsed with PBS again. Then the well was treated with 50uL of 10% Triton solution to permeabilize platelets adhering to the collagen layer. After a 20 min incubation the sample was diluted 1,000 x with PBS and then incubated for 30 min with the LDH detection solution from the Promega assay kit. The fluorescence of each sample was then determined, and the concentration calculated from a standard curve of a known concentration of permeabilized platelets. The fluorescence detected from the LDH ‘released’ by the LPs alone was also measured and deducted from each sample so only the adherence of fresh platelets was measured.

### 2.7 Platelet adhesion under flow assay

A procedure for assessing the adhesion of platelets to a collagen surface under flow conditions was developed using calcein AM live cell stain (ThermoFisher cat# C3099). First a 0.1 mg/ml fibrillar collagen solution (Chrono-Log, cat# 385) was incubated in a fluidic glass bottom channel (Ibidi, cat# 80167) for 1 h to deposit a collagen layer on which platelets could adhere. Then the microfluidic channel was gently rinsed with PBS to remove any excess collagen. Separately the platelets obtained in PRP were stained using a calcein AM live-cell stain. This live-cell stain was selected because it does not stain the LPs which cannot undergo a metabolic process. Experimental samples were created using the calcein-stained fresh platelets at 200,000 plt/uL and 75,000 plt/uL to simulate normal and thrombocytopenic platelet count conditions, respectively. For each condition a sample was made with no added LPs and with LPs added at ratios of 5:1, and 5:2 platelets to LPs. A control sample was also made with an LP concentration of 200,000 and 75,000 LPs/uL, respectively, but without fresh platelets. The lyophilized platelets used in the control were incubated with calcein AM to confirm that they did not generate a fluorescent signal. All conditions incorporated 40% hematocrit to simulate physiological conditions and corn trypsin inhibitor (Prolytix, cat# CTI-01) was also added at 50 ug/mL to inhibit the contact pathway of coagulation. Using a syringe pump (Cole-Parmer, cat# 78–0200C) the samples were then steadily pumped through the microfluidic channel at a rate of 1,800 uL/min for 4 min to produce a shear stress of 20.16 dyn/cm^2^, selected to simulate physiological shear in arteries (Malek et al., 1999). The microfluidic channels were then all rinsed with tyrode at a rate of 1,800 uL/min for 4 min to remove any platelets that had not adhered to the collagen. A second rinse of 250 uL of tyrode was conducted *via* pipetting to remove any additional excess cells. Then the channel was incubated with 100 U/mL collagenase in tyrode for 1 h at 37°C to break down the collagen layer and release the adhered platelets. Each channel was thoroughly rinsed with 250 uL of tyrode and the fluorescence of each collected sample was then measured (Ex/Em 494nm/517 nm) and normalized. Fresh platelet and LP counts for all conditions in the LTA, static adhesion, and adhesion under flow assays are listed for reference in supplementary data (Supplementary Table S3).

### 2.8 Scanning electron microscopy

A silicon wafer (Ted Pella, cat# 16006) was prepared by rinsing the wafer in ethanol and then PBS. Then a 0.1 mg/ml collagen solution (Chrono-Log, cat# 385) was incubated with the silicon wafer for 1 h to form a collagen layer. Then the excess collagen was removed and the samples, fresh platelets and LPs respectively, were incubated with the silicon wafer for 2 h before centrifuging the samples at 150 g for 5 min. The samples were subsequently fixed with a 2.5% glutaraldehyde and 1% paraformaldehyde in a 0.12 M sodium cacodylate buffer. The samples were then washed with 0.12 M sodium cacodylate buffer and fixed with 1% osmium tetroxide in a 0.12 M sodium cacodylate buffer. The samples were afterwards dehydrated with increasing EtOH concentrations and dried with hexamethyldisilane. The samples were imaged with a FEI Teneo Scanning Electron Microscope (SEM) using a low vacuum detector. The images were taken in low vacuum (10 Pa) at 5 kV voltage and 50 pA current settings.

### 2.9 Statistical analysis

All graphical representations and statistical analyses of the data were developed using the Prism software from GraphPad. Statistical significance between experimental groups in the receptor characterization, light transmittance aggregometry, and adherence assay trials was determined using an analysis of variance (ANOVA) method. A Tukey’s multiple comparison test was used for the *post hoc* analysis.

## 3 Results

### 3.1 Analysis of surface receptor expression on lyophilized platelets

**FIGURE 1:**
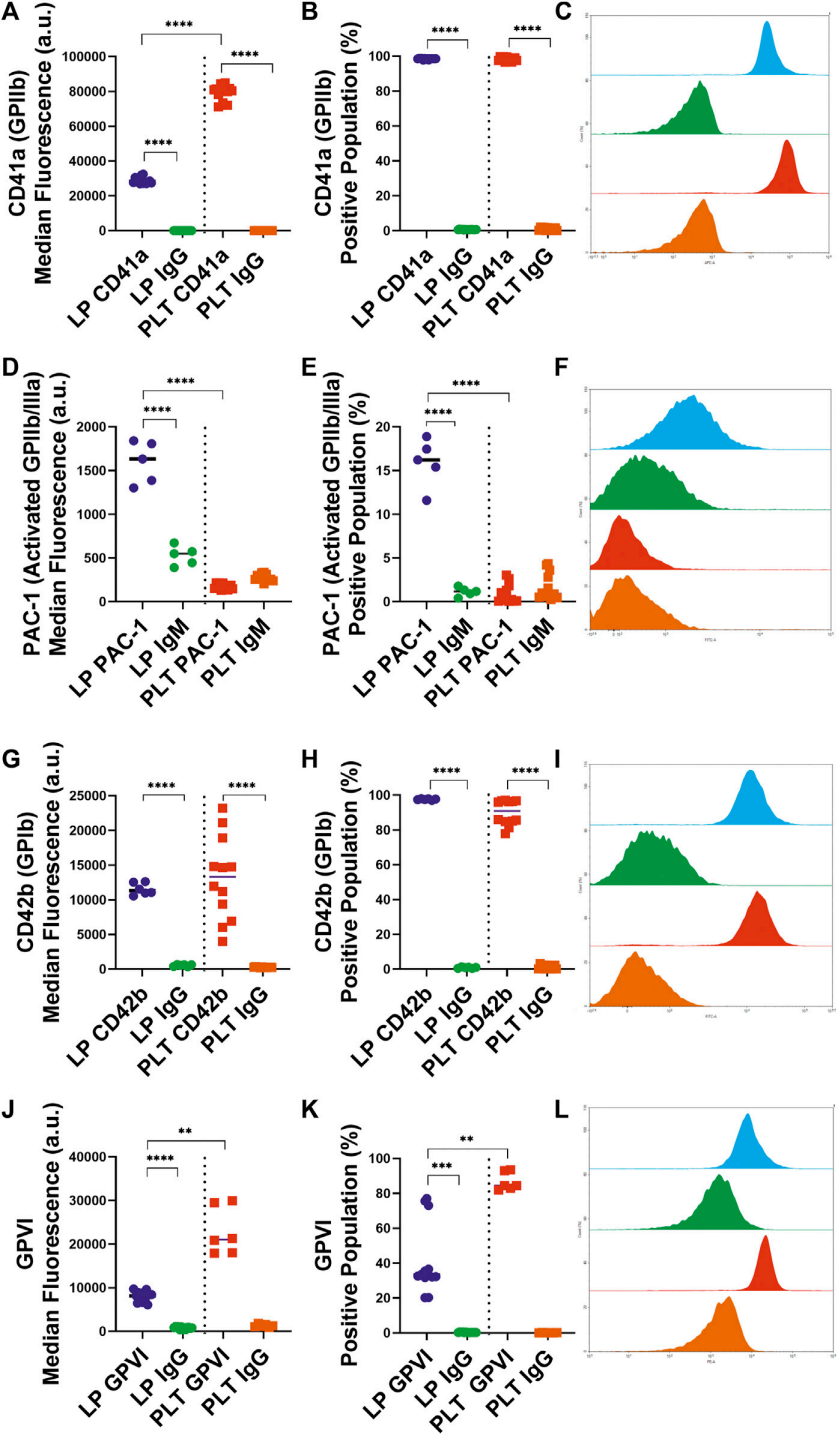
Characterization of Key Receptor Surface Expression on Lyophilized and Fresh Human Platelets by Flow Cytometry. Quantification of expression level (left), percent positive events (middle), and representative histograms (right), for **(A,B,C)** GPIIb [LP: *n* = 14, PLT: donors = 5, *n* = 12], **(D,E,F)** active GPIIb/IIIa [LP: *n* = 6, PLT: donors = 4, *n* = 12], **(G,H,I)** GPIb [LP: *n* = 6, PLT: donors = 5, *n* = 12], (J,K,L) GPVI [LP: *n* = 13, PLT: donors = 3, *n* = 6].

To assess the interactions of the LPs with untreated native platelets, the availability of key receptors was characterized. Almost all (98.4%) LPs retained approximately one-third of GPIIb (CD41a) at their surface compared to platelets with median fluorescence values of 28,348 versus 80,556 a. u. (*p* < 0.0001), respectively (Figures 1A–C). Next, we determined the fraction of activated GPIIb/IIIa receptors using a PAC-1 antibody. 15.8% of the LP population exhibited the active conformation of the GPIIb/IIIa receptor (Figures 1D–F). Thus, the activated receptor that is critical to the platelet aggregation process and the late stages of adhesion on thrombogenic surfaces is available on some of the LP carriers. After analyzing the GPIIb receptor availability, the presence of the GPIb (CD42b) receptor essential for surface adhesion was characterized. In contrast with GPIIb, LPs retained the majority of GPIb receptors compared to platelets (Figures 1G–I). Additionally, a significant portion of the LPs (41.0%, Figure 1K), maintained expression of the GPVI receptor (*p* = 0.0042). Though this expression was reduced from platelets in terms of both percentage of the positive population and median fluorescence (Figures 1J–L; *p* = 0.0042 and *p* = 0.0062, respectively).

### 3.2 Analysis of phosphatidylserine exposure and fibrinogen binding to lyophilized platelets

**FIGURE 2:**
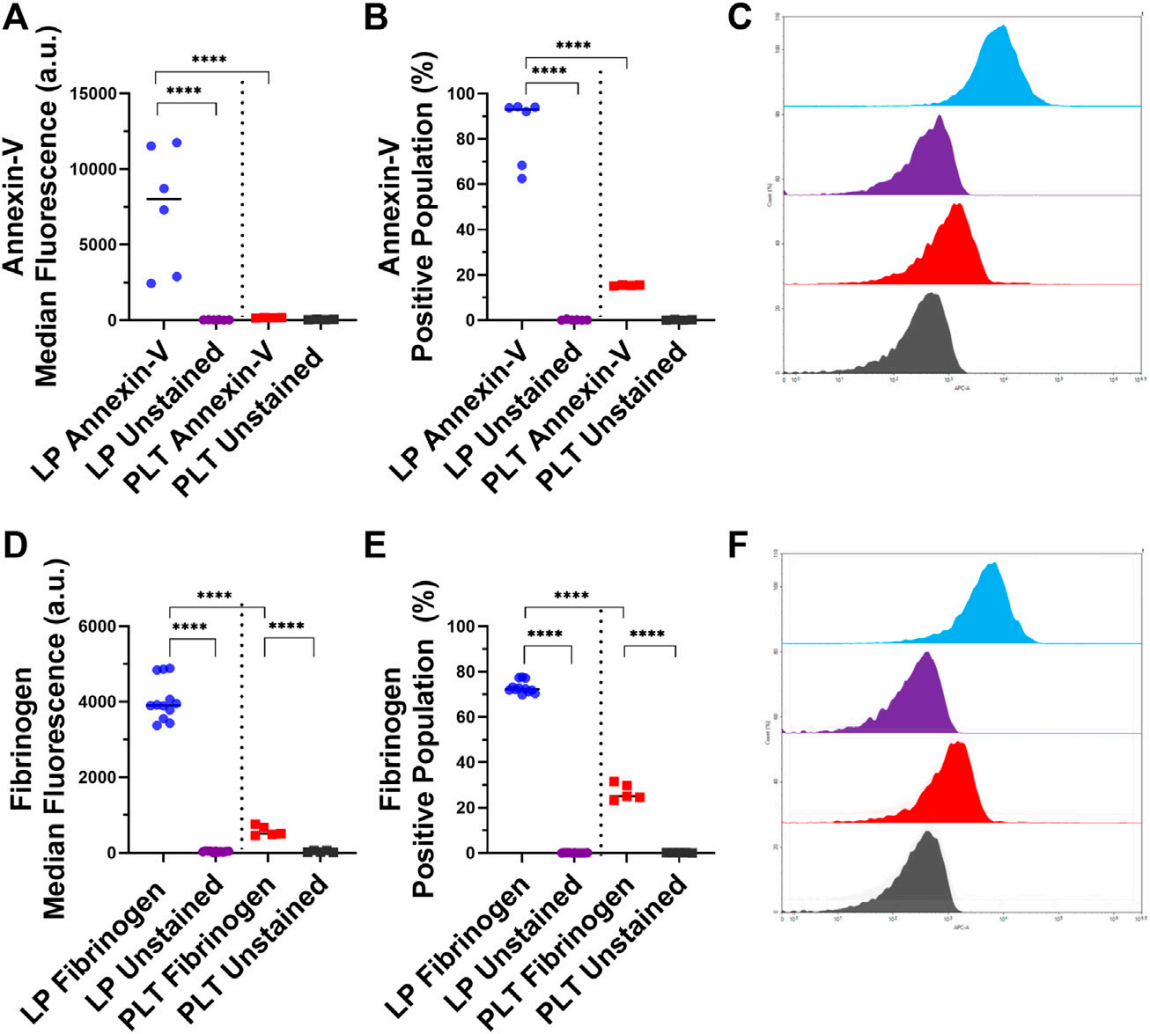
Characterization of Phosphatidylserine Exposure and Fibrinogen Binding on Lyophilized and Fresh Human Platelets. Quantification of expression level (left), percent positive events (middle), and representative histograms (right), for **(A,B,C)** phosphatidylserine [LP: *n* = 6, PLT: donors = 2, *n* = 4], and **(D,E,F)** fibrinogen binding [LP: *n* = 12, PLT: donors = 2, *n* = 5].

After analyzing the key platelet receptors on the lyophilized and control platelets, we subsequently characterized the pro-coagulant characteristics of the LPs. One of the most important markers for pro-coagulant platelets is the presence of exposed phosphatidylserine on activated platelets, which is involved in the regulation of fibrin formation during thrombosis (Reddy and Rand, 2020). A large majority of LPs (84.2%) displayed significantly more exposed phosphatidylserine compared the control platelets (Figures 2A–C; *p* < 0.0001).

Next, the binding of fibrinogen to the surface of the LPs was assessed due to the central role of fibrinogen in platelet aggregation and thrombogenesis. The flow cytometry results demonstrated that the LPs had 7.7 times more binding of fibrinogen compared to the control platelets (Figure 2D; *p* < 0.0001). The percentage of fibrinogen binding in these two populations was also significantly different: 73.1 and 26.8% for LPs and platelets, respectively (Figure 2E; *p* < 0.0001).

### 3.3 Aggregation of platelets with different ratios of lyophilized platelets

**FIGURE 3:**
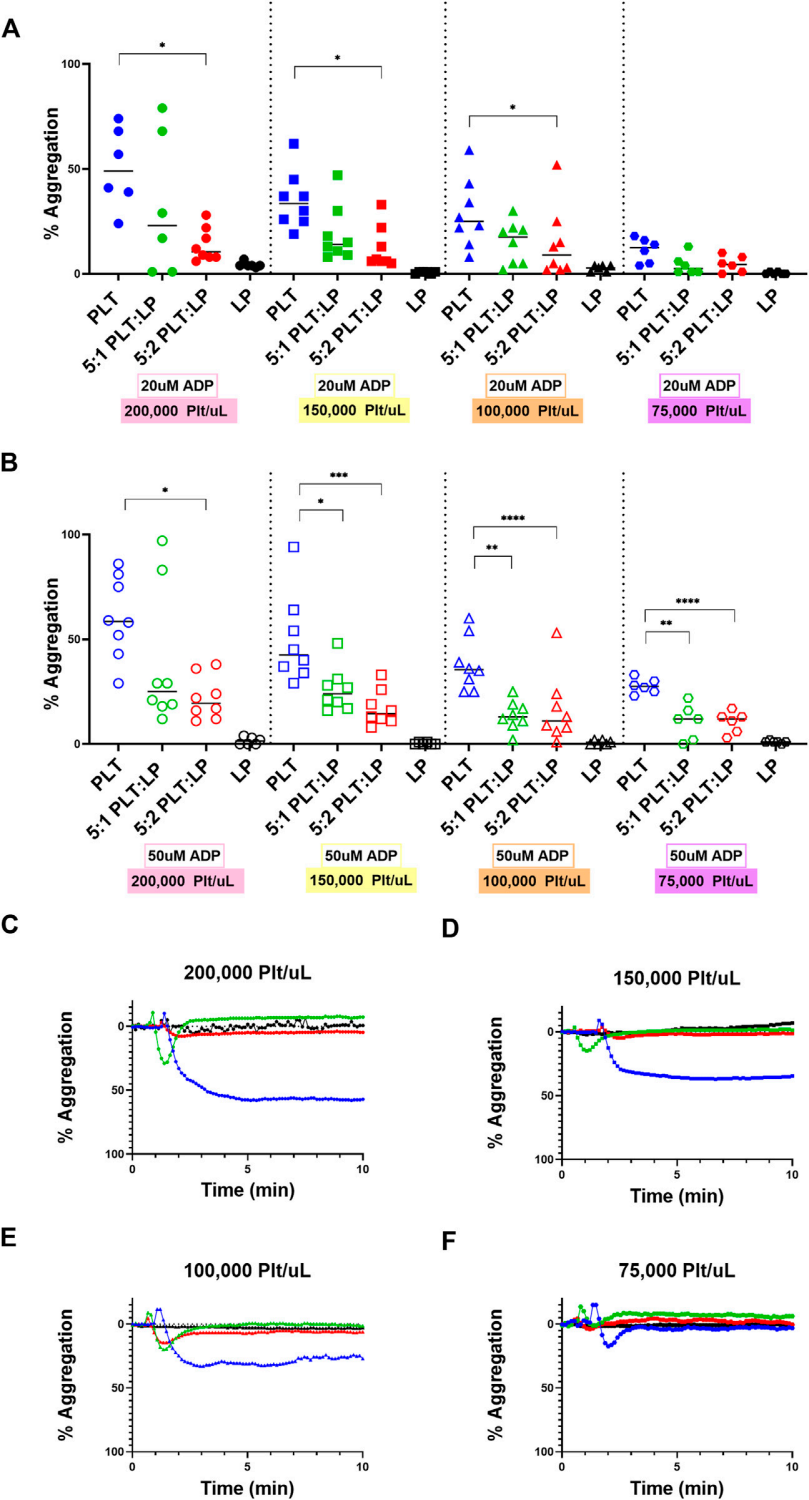
Aggregation of Platelets in the presence of LPs at Different Concentrations and Ratios. Quantification **(A,B)** and representative LTA curves **(C–F)** for the aggregation of platelets compared to platelets mixed with LPs at ratios of 5:1 and 5:2 platelets to LPs using ADP agonist at concentrations of 20 uM (A; shown in representative figures **(C–F)** and 50 uM **(B)** using healthy platelet levels of **(C)** 200,000 platelets/uL [donors = 4, *n* = 8] and **(D)** 150,000 platelets/uL [donors = 4, *n* = 8], as well as thrombocytopenic platelet levels of **(E)** 100,000 platelets/uL [donors = 4, *n* = 8] and **(F)** 75,000 platelets/uL [donors = 3, *n* = 6] for all ratios. LP alone conditions are included for each platelet condition as a control.

With the most significant platelet interaction receptors characterized, the effect of LPs on platelet aggregation was analyzed using LTA. This analysis was conducted with a ratio of 5:1 and 5:2 platelets to LPs in order to approximate the ratios achieved with one and two standard platelet transfusions and compare to the 5:0 platelet control. These experiments were also tested at different platelet count conditions ranging from thrombocytopenic scenarios (75,000 and 100,000 platelets/uL) to normal healthy counts (150,000 and 200,000 platelets/uL). ADP was used as a platelet agonist. In each condition, there was a significant decrease in platelet maximum aggregation between the fresh platelets alone and the fresh platelets mixed with LPs. The trend was consistent across all platelet concentrations and with two different concentrations of platelet agonists (Figures 3A–F). Specifically, at the normal platelet concentration of 200,000 Plt/uL, there was a significant decrease in platelet maximum aggregation with the 5:2 platelet:LP ratio for both ADP concentrations (Figures 3A–C; *vs.* 20uM: *p* = 0.0426, *vs.* 50 uM: *p* = 0.0158). This effect was also observed with the alternative normal platelet concentration of 150,000 Plt/uL with the 5:2 ratio at both ADP agonist concentrations (Figures 3A,B,D; *vs.* 20 uM: *p* = 0.0180, *vs.* 50 uM: *p* = 0.0003). Under thrombocytopenic conditions there was a significant decrease in maximum aggregation for both agonist concentrations when 100,000 Plt/uL were mixed with LPs at a 5:2 ratio (Figures 3A,B,E; *vs.* 20 uM: *p* = 0.0444, *vs.* 50 uM: *p* = 0.0002), but this same trend only appears at the 50 uM ADP agonist concentration for the 5:2 ratio sample in the 75,000 Plt/uL condition (Figures 3B,F; *p* < 0.0001). Furthermore, a significant inhibitory effect was seen at the higher agonist condition and 5:1 ratio for 150,000 Plt/uL, 100,000 Plt/uL, and 75,000 Plt/uL conditions (Figure 3B; *p* = 0.0133, *p* = 0.003, and *p* = 0.0001, respectively). This trend was also visible in most conditions, but it was only statistically significant at the high agonist concentration. There also appears to be a dose dependent effect with the higher dose of LPs resulting in lower aggregation, but this was not statistically significant.

### 3.4 Adhesion of platelets in mixtures with lyophilized platelets

**FIGURE 4:**
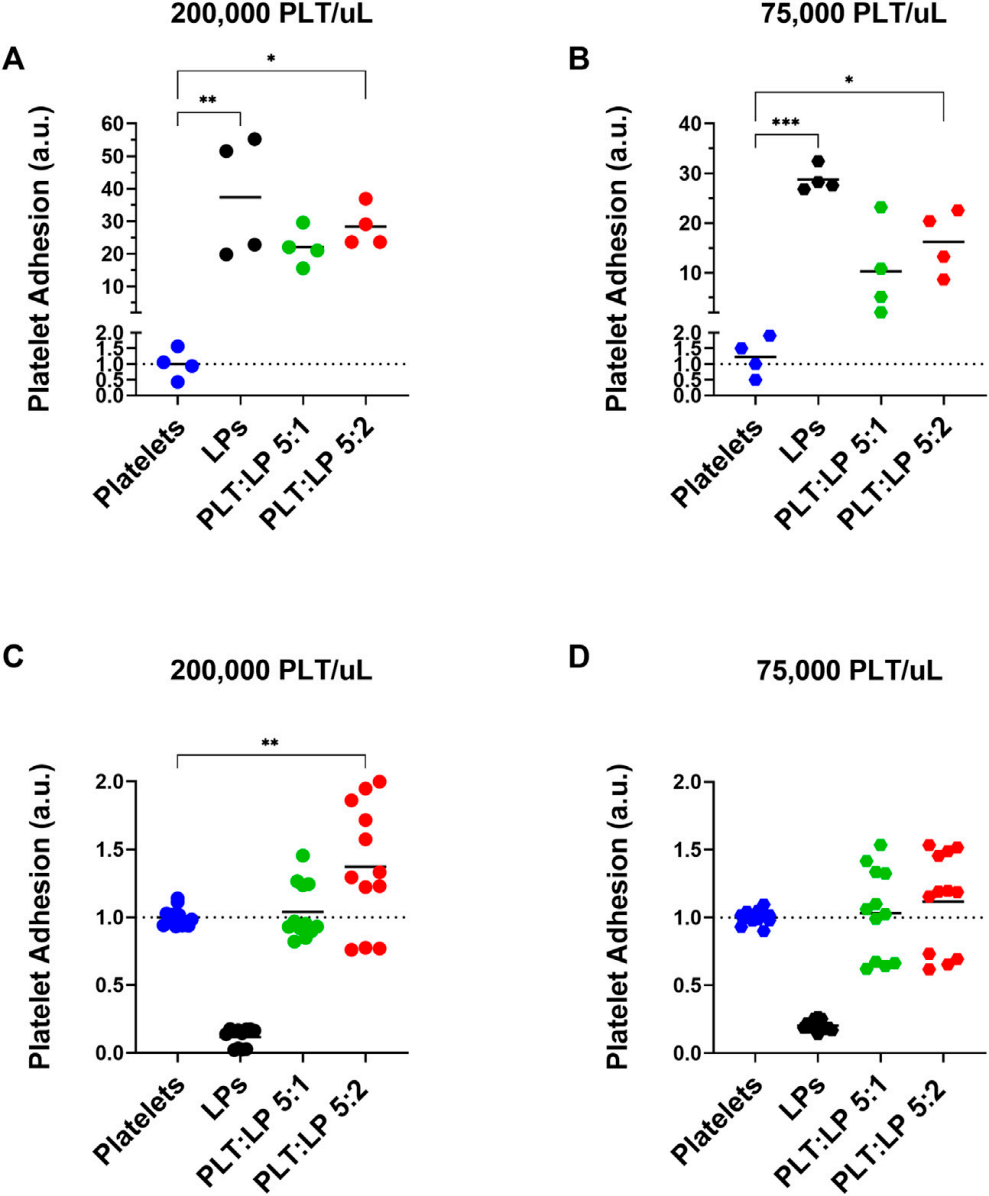
Adhesion of Platelets Mixed with Lyophilized Platelets on a Collagen Surface. Normalized quantification of the adhesion of platelets in comparison to platelets mixed with LPs (at ratios of 5:1 and 5:2 platelets to LPs) in static conditions using and LDH assay **(A,B)** and under flow using calcein AM live-cell staining **(C,D)**. Both assays were conducted at a **(A,C)** normal platelet concentration of 200,000 platelets/uL [Static: donors = 2, *n* = 4, Flow: donors = 3, *n* = 12], and a **(B,D)** thrombocytopenic platelet concentration of 75,000 platelets/uL [Static: donors = 2, *n* = 4, Flow: donors = 3, *n* = 12]. Note that the calcein AM live-cell staining **(C,D)** does not stain the inert LPs and thus the LP only condition does not represent their adhesion and is used as a control to ensure that there is no off target staining.

The other aspect of platelet function that needed to be examined was the adhesion of the platelets to a thrombogenic surface. During clot formation, platelets adhere to exposed collagen at an injury site and become activated. This initiates the formation of a platelet plug and is an essential aspect of hemostasis. To assess the effect of LPs on this key platelet function, the adhesion of the platelets to a collagen-coated surface while in a mixture with LPs was assessed under static and flow conditions. The adhered platelets under static conditions were measured through a LDH fluorescence assay. Appropriate controls were also conducted to account for the contributions of LPs to the measured fluorescence. This was repeated under healthy and thrombocytopenic conditions to simulate different scenarios where LPs could interact with native platelets. LPs adhered significantly more to the collagen surface compared to platelets in both normal (Figure 4A, 37.4-time more, *p* = 0.0015) and thrombocytopenic scenarios (Figure 4B, 28.8-time more, *p* = 0.0001). The results also showed a significant increase in platelet adhesion when mixed with LPs at both healthy (Figure 4A, 28.3-time increase, *p* = 0.0123) and thrombocytopenic conditions (Figure 4B, 16.2-time increase, *p* = 0.0153) at a 5:2 ratio. This indicates that the LPs interact with untreated platelets to promote surface adhesion. There appears to be a slight but not statistically significant dose effect for the 5:1 and 5:2 platelet to LP ratios as well.

The adhesion of the platelets was also assessed under flow using calcein AM-stained platelets in a microfluidic channel. The calcein AM live-cell stain was chosen because it does not stain the metabolically inactive LPs, and thus there is no LP component to the detected fluorescence (Figures 4C,D). The adherence was measured using a platelet control as well as platelets mixed with LPs at a ratio of 5:1 and 5:2 platelets to LPs for both normal and thrombocytopenic conditions. Compared to the fresh platelet control, there was a significant increase in the adherence of platelets under flow for those mixed with LPs at a 5:2 ratio with normal platelet counts (Figure 4C, 1.4-time increase, *p* = 0.0042). A similar trend was seen for the 5:2 platelet to LP ratio under thrombocytopenic conditions, though the difference was not significant (Figure 4D, 1.1-time increase, *p* = 0.6384). This indicates that the LPs promote surface adhesion of fresh platelets even under shear stress from flow conditions. Though, the pro-adhesion effect is small under flow compared to static conditions.

## 4 Discussion

The objective of this study was to characterize the interactions between LPs and untreated platelets. The scanning electron microscopy observation of platelets versus LPs on a collagen surface showed that LPs have much less pseudopodia than the adhered platelets (Supplementary Figures S1A,B), which is consistent with the fact that LPs are fixed and unable to undergo further morphological changes. Though these images provide some interesting insight into the possible morphological changes, quantitative analyses of the platelet receptors and functions is needed to understand differences between LPs and fresh platelets. The first aspect of this analysis was the characterization of the receptors available on the surface of the LPs. The presented data demonstrates that almost all the LPs do maintain two critical receptors for platelet interactions and adhesion, GPIIb and GPIb. The GPIIb/IIIa receptor is essential for the aggregation of platelets through binding to fibrinogen and von Willebrand Factor. The conservation of this receptor on the LPs (Figures 1A–C) is a strong indicator that these lyophilized platelets can, at least weakly, interact with untreated platelets or any GPIIb/IIIa ligands. Furthermore, a significant percentage, 15.8%, of these LPs have GPIIb/IIIa receptors in the active conformation (Figures 1D–F). The activation of these LPs is likely due to the temperature decrease in the lyophilization process since cold exposure is known to cause platelet activation (Gousset et al., 2004). This activated GPIIb/IIIa receptor increases the ability of the LPs to interact with its ligands (e.g. fibrinogen) *via* enhanced affinity (Michelson, 2013). Additionally, the primary receptor for platelet adhesion, GPIb, is also significantly maintained on the LP surface (Figures 1G–I). A significant portion of the LPs also retain the GPVI receptor important for collagen binding (Figures 1J–L). Thus, the LPs can be expected to interact with thrombogenic surfaces (Figures 4A–D). This characterization of the surface receptors present in the LPs provides strong evidence that the LPs can interact with native platelets and affect their performance in aggregation and adhesion.

This initial receptor data was then reinforced by assessing the exposure of phosphatidylserine on the LPs, as well as the fibrinogen binding to the LPs. The exposed phosphatidylserine on platelets has been shown to regulate fibrin binding and thrombogenesis and thus serves as a marker to identify pro-coagulant platelets (Reddy and Rand, 2020). As seen in Figure 2, the LPs have a significant annexin-V staining which indicates significantly more exposed phosphatidylserine compared to unaltered platelets (Figures 2A–C). Additionally, the fibrinogen binding to the LPs is shown to be significantly increased compared to the unaltered platelets (Figures 2D–F). This correlates with the expression of activated GP IIb/IIIa receptors detected on the surface of the LPs since these activated receptors are known to have an increased affinity for fibrinogen (Michelson, 2013). Given the key role of fibrinogen in platelet interaction and thrombosis, the increased fibrinogen binding also indicates that these LPs will significantly interact with unaltered platelets and may have a pro-hemostatic effect.

The effects of LPs on the aggregation and adhesion of untreated platelets were then assessed through the LTA, LDH, and calcein AM assays. The evidence clearly demonstrates that these LPs do interact with the untreated platelets and have a significant impact on their function. However, it is interesting to note that interactions have contrary effects for aggregation versus adhesion. The LPs have a robust anti-platelet effect in terms of aggregation, as we demonstrated that this effect is seen at extremely high ADP concentrations (including 50 uM). Indeed, there were significant decreases in platelet aggregation when LPs were mixed with the untreated platelets. This was a consistent trend across platelet counts as well. Additionally, our supplementary data (Supplementary Figure S2) indicates that there are no factors in the centrifuged PPP that could account for the observed anti-platelet effect. Thus, the data demonstrates that LPs inhibit the aggregation function of untreated platelets. In addition, Bynum *et al.* observed that the maximum clot firmness is significantly reduced in presence of LPs (Bynum et al., 2019). Our observation of the antiplatelet effect on fresh platelet aggregation (Figure 3) could potentially explain this reduced clot firmness. However, the LPs have an equally significant pro-platelet effect in terms of adhesion. There was an increase in the adherence of platelets to a collagen surface under static conditions when untreated platelets were mixed with LPs. This phenomenon offers an interesting contrast to the effect of LPs on aggregation. The evidence shows that the LPs improve the ability of platelets to interact and adhere with a surface while interfering with the ability of platelets to aggregate. In addition, our data corroborate the findings by J.A. Bynum *et al.* and G. M. Fitzpatrick *et al.* demonstrating that LPs adhere to collagen thrombogenic surface (Bynum et al., 2019), despite a reduction in GPIb expression after they undergo freeze drying (Fitzpatrick et al., 2013). Moreover, LPs adhere to collagen surfaces despite them being unable to undergo firm adhesion, subsequent to a lack of signaling convergence toward the recruitment of receptors beyond GPIb/V/IX. The low GPIIb and GPVI expression might be overcome by the priming of a fraction of these platelets into an activated state by induction of GPIIb/IIIa active conformation during lyophilization. Activated GPIIb/IIIa has a high binding affinity for von Willibrand factor, which interacts with collagen, thus facilitating the consolidation of platelet adhesion by active GPIIb/IIIa receptors in support of GPIb initial tethering. Indeed, PAC-1 antibody, that specifically targets the active conformation of GPIIb/IIIa, binds LPs significantly more than untreated platelets (Figures 1D–F). Additionally, other receptors, such as α2β1 are also involved in the consolidation step of platelet adhesion on a collagen surface (Varga-Szabo et al., 2008), and might also compensate for the decreased expression of GPVI and GPIIb in LPs. Indeed, we observed promotion of platelet adhesion on thrombogenic surface in static conditions and in the presence of LPs both in normal (Figure 4A) and thrombocytopenic samples (Figure 4B). Moreover, this pro-platelet effect on adhesion was also observed under shear stress in a microfluidic channel for the 5:2 ratio of platelets to LPs at a normal platelet concentration (Figure 4C). Though the effect was greatly reduced compared to the static conditions. There was also a pro-platelet adhesion trend in the thrombocytopenic condition under flow at this ratio (Figure 4D), but it was not significant in our experimental settings. This is in contrast with the study by Bynum *et al.* that observed this effect solely in thrombocytopenic condition (Bynum et al., 2019). This could be explained by the difference in formulation between the fixed LPs used in our study and their trehalose stabilized LPs. Our results under flow conditions demonstrate that the LPs have a robust effect promoting fresh platelet adhesion, even though there is a reduction in effect compared to static adhesion.

These contrary effects for different aspects of platelet functions highlight the need for further research into the underlying mechanisms. Understanding these mechanisms could provide insight into platelet function and allow for more targeted treatments of platelet disorders and pathologies involving platelet cooperation.

The characterization of these interactions provides for interesting opportunities in therapeutics and drug delivery. The contrary effect indicates that the LPs may be used as a therapeutic that inhibits improper platelet aggregation in circulation, without hindering platelet adhesion at an injury site. For example, the LPs could be developed into a therapeutic that reduces platelet interactions with cancer cells or inhibits Tumor Cell Induced Platelet Aggregation (TCIPA) without disrupting other key platelet functions. Alternatively, the tendency of the LPs to adhere to collagen surfaces and promote platelet adhesion could be used to deliver a therapeutic to an injury site while mitigating the possibility of such an agent inducing platelet aggregation systemically.

It is possible that the activated LPs may increase the risk of thrombosis. However, in an early phase I clinical trial, there has been no sign of thrombotic complication reported to date (Ohanian et al., 2022). The antiplatelet effect of LPs on platelet aggregation observed in our study could be a possible explanation for the lack of thrombotic side effects in spite of its ability to act as hemostatic agent.

Further understanding the effects of LPs on untreated platelets would form a foundation for creating a LP therapeutic or drug delivery product. Regardless of the targeted application, an in-depth characterization of their efficiency along with limitations and potential side effects (e.g. adverse events such as thrombotic complication and dose-limiting toxicities) will be required in the context of each pathological scenario to assess risks versus benefits.

## Data Availability

The original contributions presented in the study are included in the article/Supplementary Material, further inquiries can be directed to the corresponding author.

## Glossary

ADP: Adenosine Diphosphate
CTC: Circulating Tumor Cell
FSC: Forward Scatter
GP: Glycoprotein
LDH: Lactate Dehydrogenase
LP: Lyophilized Platelets
LTA: Light Transmission Aggregometry
SEM: Scanning Electron Microscopy
SSC: Side Scatter
TCIPA: Tumor Cell Induced Platelet Aggregation
