# Supplementary material for: Lyophilized platelets inhibit platelet aggregation with simultaneous paradoxical promotion of platelet adhesion
Source: Front Bioeng Biotechnol. 2022 Aug 19;10:941817. doi: 10.3389/fbioe.2022.941817 (PMC9437314; doi:10.3389/fbioe.2022.941817)
Supplement: Supplementary file 1 [file DataSheet1.PDF]

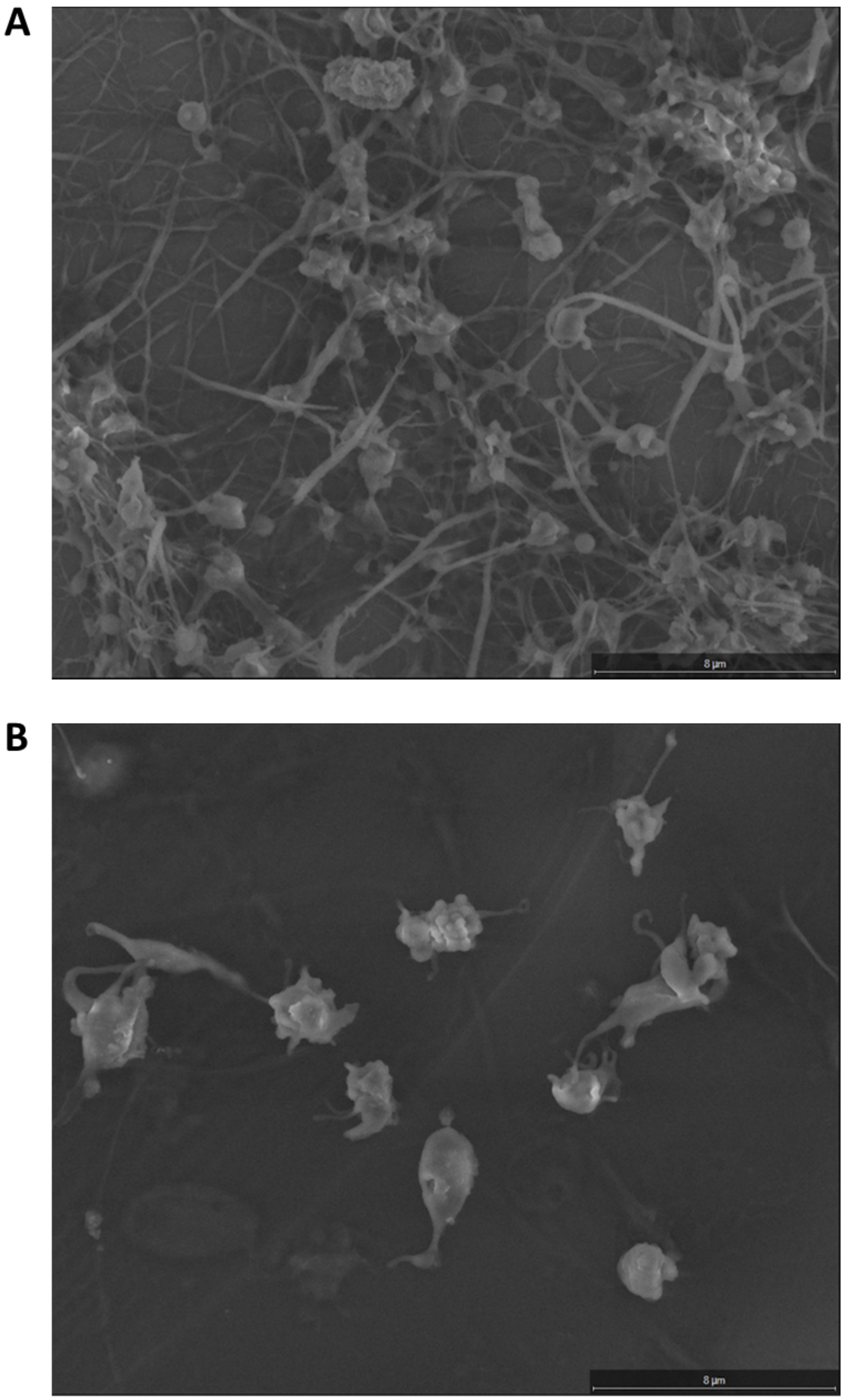

Fig. S1: Micrographs of Fresh and Lyophilized Platelets. Scanning Electron Microscopy images of (A) fresh platelets and (B) LPs on a silicon wafer with a collagen surface. The images were taken in low vacuum (10 Pa) at 5 kV voltage and 50pA current settings.

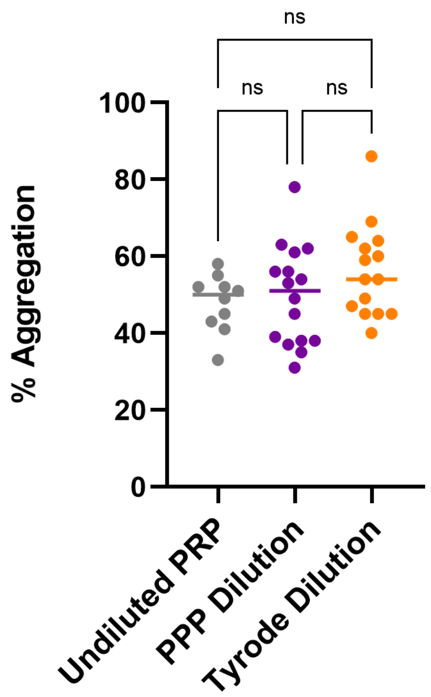

Fig. S2: Aggregation of PRP compared to Diluted PRP. A LTA analysis of platelet aggregation comparing the aggregation of platelets in undiluted PRP (250,000 platelet/uL and 370,000 platelet/uL) (left, 2 donors, n=10 replicates) with the aggregation of platelets in PRP diluted to 200,000 platelets/uL with PPP obtained *via* centrifugation (middle, 2 donors, n=16 replicates) with tyrode buffer (right, 2 donors, n=15 replicates).

Aggregation Assay						
200,000 PLT/uL condition	PLT Concentration PLT/uL	LP Concentration LP/uL	Total Volume uL	Total PLT Count	Total LP Count	Total PLT + LP
5:0; PLT:LP	200,000	0	250	50,000,000	0	50,000,000
5:1; PLT:LP	200,000	40,000	250	50,000,000	10,000,000	60,000,000
5:2; PLT:LP	200,000	80,000	250	50,000,000	20,000,000	70,000,000
LP control	0	200,000	250	0	50,000,000	50,000,000
150,000 PLT/uL condition	PLT Concentration PLT/uL	LP Concentration LP/uL	Total Volume uL	Total PLT Count	Total LP Count	Total PLT + LP
5:0; PLT:LP	150,000	0	250	37,500,000	0	37,500,000
5:1; PLT:LP	150,000	30,000	250	37,500,000	7,500,000	45,000,000
5:2; PLT:LP	150,000	60,000	250	37,500,000	15,000,000	52,500,000
LP control	0	150,000	250	0	37,500,000	37,500,000
100,000 PLT/uL condition	PLT Concentration PLT/uL	LP Concentration LP/uL	Total Volume uL	Total PLT Count	Total LP Count	Total PLT + LP
5:0; PLT:LP	100,000	0	250	25,000,000	0	25,000,000
5:1; PLT:LP	100,000	20,000	250	25,000,000	5,000,000	30,000,000
5:2; PLT:LP	100,000	40,000	250	25,000,000	10,000,000	35,000,000
0:5 PLT:LP	0	100,000	250	0	25,000,000	25,000,000
75,000 PLT/uL condition	PLT Concentration PLT/uL	LP Concentration LP/uL	Total Volume uL	Total PLT Count	Total LP Count	Total PLT + LP
5:0; PLT:LP	75,000	0	250	18,750,000	0	18,750,000
5:1; PLT:LP	75,000	15,000	250	18,750,000	3,750,000	22,500,000
5:2; PLT:LP	75,000	30,000	250	18,750,000	7,500,000	26,250,000
0:5 PLT:LP	0	75,000	250	0	18,750,000	18,750,000
Static Adhesion Assay						
200,000 PLT/uL condition	PLT Concentration PLT/uL	LP Concentration LP/uL	Total Volume uL	Total PLT Count	Total LP Count	Total PLT + LP
5:0; PLT:LP	200,000	0	100	20,000,000	0	20,000,000
5:1; PLT:LP	200,000	40,000	100	20,000,000	4,000,000	24,000,000
5:2; PLT:LP	200,000	80,000	100	20,000,000	8,000,000	28,000,000
0:5 PLT:LP	0	200,000	100	0	20,000,000	20,000,000
LP control (5:2)	0	80,000	100	0	8,000,000	8,000,000
LP control (5:1)	0	40,000	100	0	4,000,000	4,000,000
75,000 PLT/uL condition	PLT Concentration PLT/uL	LP Concentration LP/uL	Total Volume uL	Total PLT Count	Total LP Count	Total PLT + LP
5:0; PLT:LP	75,000	0	100	7,500,000	0	7,500,000
5:1; PLT:LP	75,000	15,000	100	7,500,000	1,500,000	9,000,000
5:2; PLT:LP	75,000	30,000	100	7,500,000	3,000,000	10,500,000
0:5 PLT:LP	0	75,000	100	0	7,500,000	7,500,000
LP control (5:2)	0	30,000	100	0	3,000,000	3,000,000
LP control (5:1)	0	15,000	100	0	1,500,000	1,500,000
Adhesion Under Flow Assay						

200,000 PLT/uL condition	PLT Concentration PLT/uL	LP Concentration LP/uL	Total Volume uL	Total PLT Count	Total LP Count	Total PLT + LP
5:0; PLT:LP	200,000	0	7200	1,440,000,000	0	1,440,000,000
5:1; PLT:LP	200,000	40,000	7200	1,440,000,000	288,000,000	1,728,000,000
5:2; PLT:LP	200,000	80,000	7200	1,440,000,000	576,000,000	2,016,000,000
0:5 PLT:LP	0	200,000	7200	0	1,440,000,000	1,440,000,000
75,000 PLT/uL condition	PLT Concentration PLT/uL	LP Concentration LP/uL	Total Volume uL	Total PLT Count	Total LP Count	Total PLT + LP
5:0; PLT:LP	75,000	0	7200	540,000,000	0	540,000,000
5:1; PLT:LP	75,000	15,000	7200	540,000,000	108,000,000	648,000,000
5:2; PLT:LP	75,000	30,000	7200	540,000,000	216,000,000	756,000,000
0:5 PLT:LP	0	75,000	7200	0	540,000,000	540,000,000

Table S3: Detailed Platelet and LP Concentrations used for Each Mixing Experiment. To clarify the procedure used for experiment involving mixing untreated platelets and LPs in different ratios, this table includes the untreated platelet and LP concentration and total cell number added to each respective sample for both normal and thrombocytopenic conditions.
